# Supplementary figures and images for: Loss of CRMP2 O-GlcNAcylation leads to reduced novel object recognition performance in mice
Source: Open Biol. 2019 Nov 27;9(11):190192. doi: 10.1098/rsob.190192 (PMC6893399; doi:10.1098/rsob.190192)

A

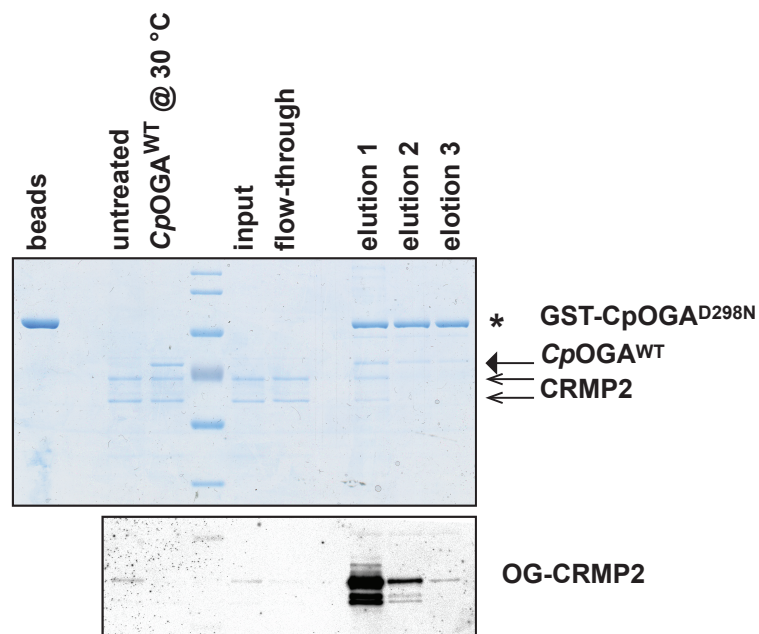

B

|        | [NH <sub>3</sub> ] <sup>+</sup> | V>     | <T     | T>     | <P     | P>     | <A     | A>     | <s      | s> | <S | S> | <A | A> | <K | K-(COOH) |
|--------|---------------------------------|--------|--------|--------|--------|--------|--------|--------|---------|----|----|----|----|----|----|----------|
| a (+1) | 74.06                           | 173.13 | 274.18 | 371.23 | 442.27 | 732.38 | 819.41 | 890.45 | 1018.54 |    |    |    |    |    |    |          |
| c (+1) | 119.08                          | 218.15 | 319.20 | 416.25 | 487.29 | 777.40 | 864.43 | 935.47 | 1063.56 |    |    |    |    |    |    |          |
| y (+1) | 963.50                          | 864.43 | 763.38 | 666.33 | 595.29 | 305.18 | 218.15 | 147.11 | 1064.55 |    |    |    |    |    |    |          |
| z (+1) | 948.49                          | 849.42 | 748.37 | 651.32 | 580.28 | 290.17 | 203.14 | 132.10 | 1049.54 |    |    |    |    |    |    |          |

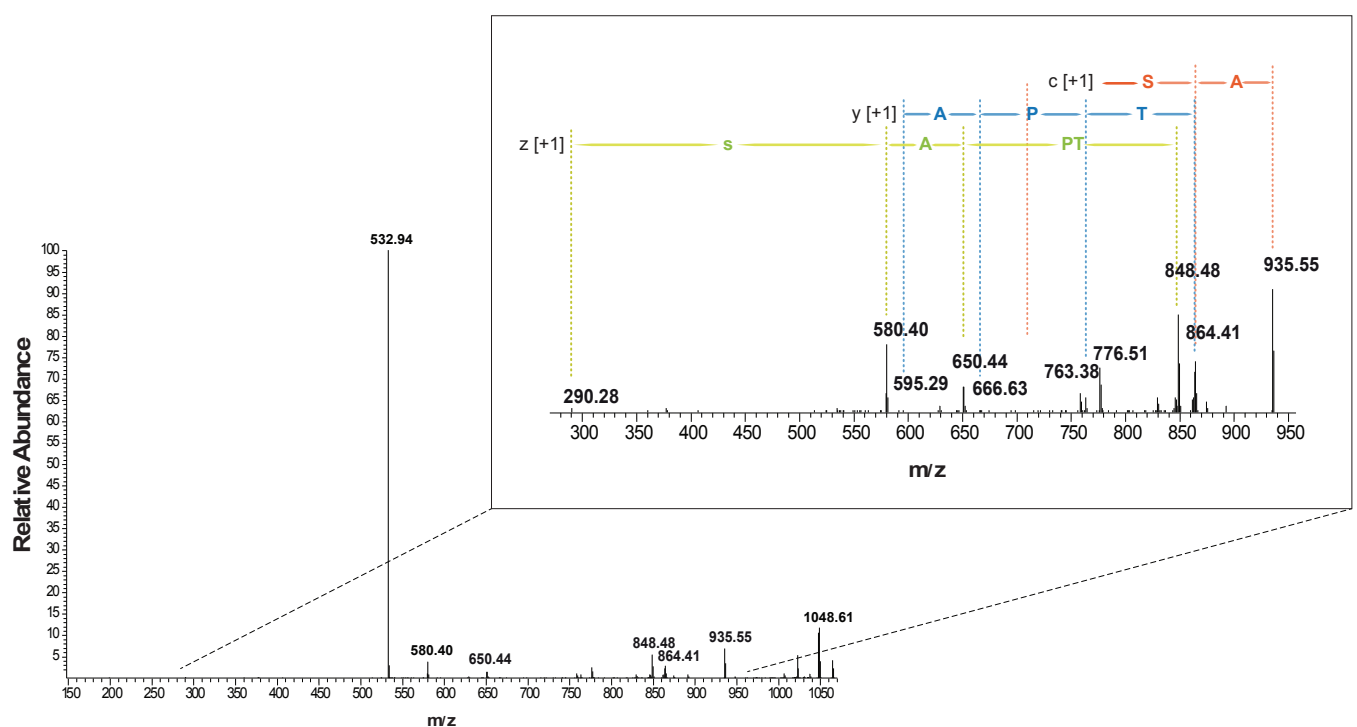

Supplementary Figure S1.

Supplement: Figure S1: Purification and site mapping of O-GlcNAc CRMP2 [file rsob190192supp1.pdf]

**A**

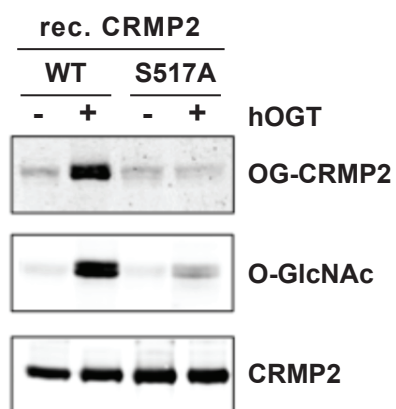

**B**

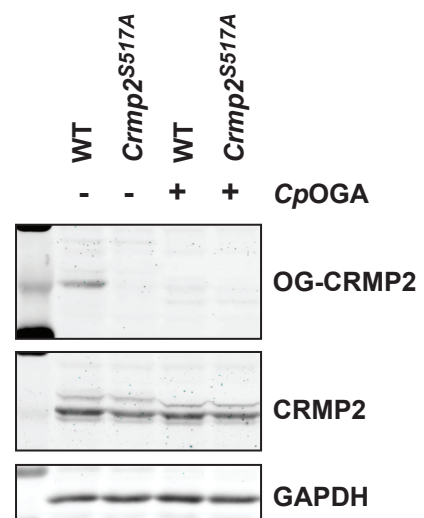

Supplementary Figure S2.

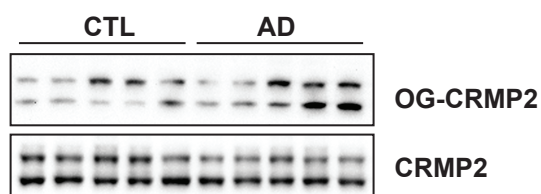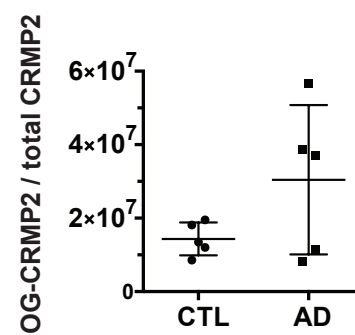

Supplementary Figure S3.

Supplement: Figure S2: Site-specific O-GlcNAc-CRMP2 antibody (OG-CRMP2) recognises Ser517 O-GlcNAcylated CRMP2 in vitro and in vivo.; Figure S3 [file rsob190192supp2.pdf]

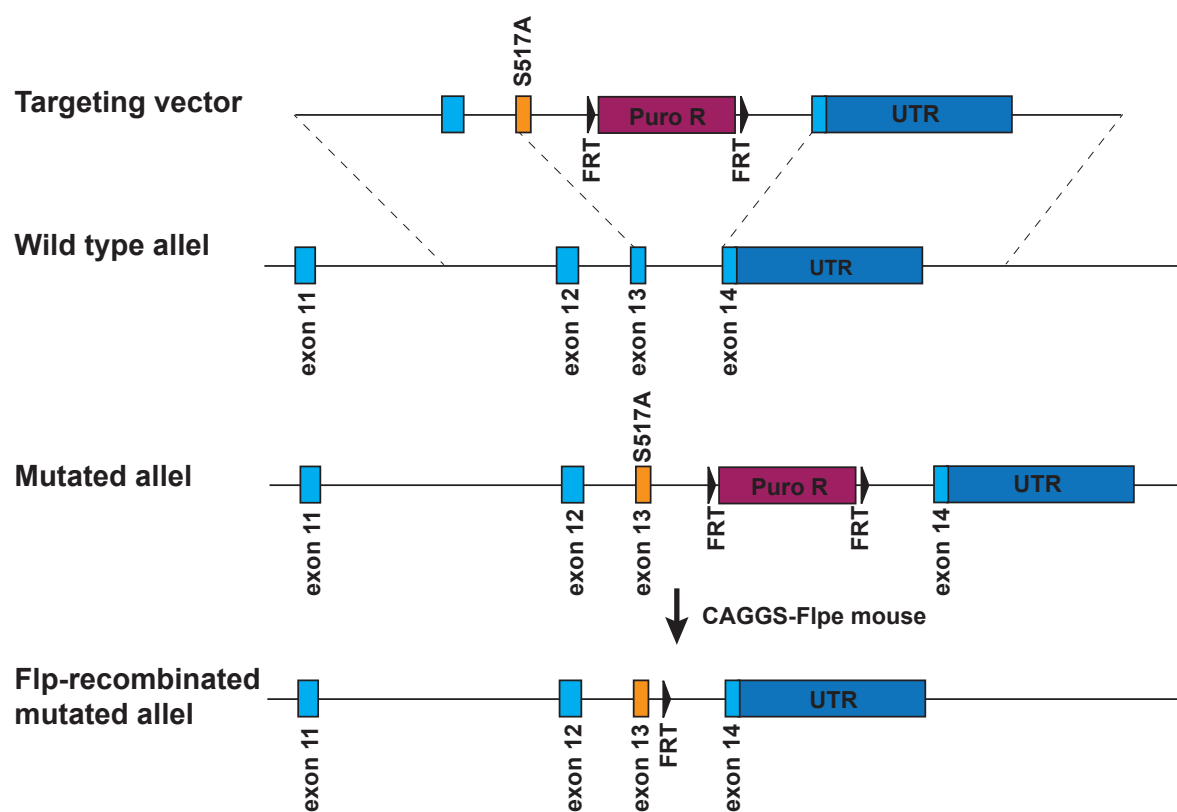

**Supplementary Figure S4.**

Supplement: Figure S4: Generation of Crmp2S517A mice lacking O-GlcNAcylation site. [file rsob190192supp3.pdf]

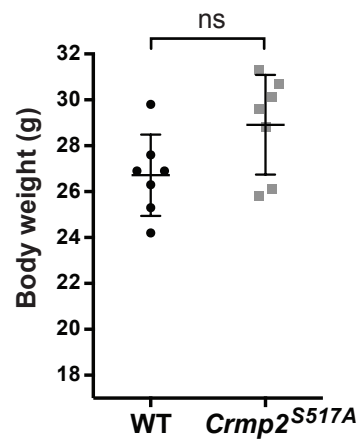

Supplementary Figure S5.

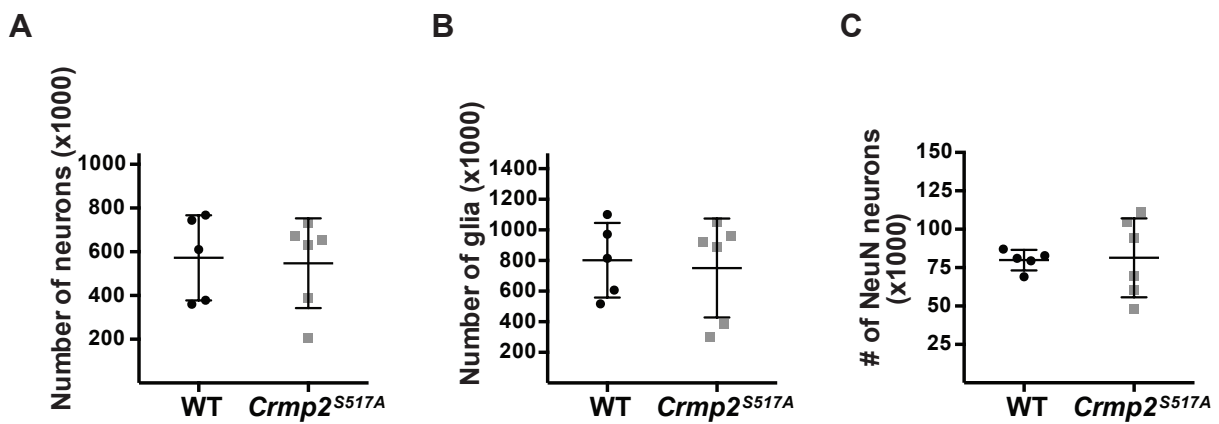

Supplementary Figure S6.

Supplement: Figure S5: Crmp2S517A mice in 87.5% BL57/6j genetic background show no alteration in weight.; Figure S6 [file rsob190192supp4.pdf]

**A**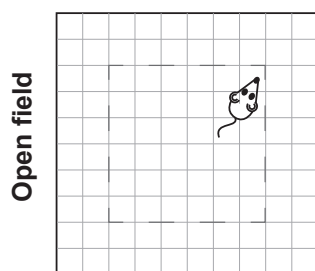**B**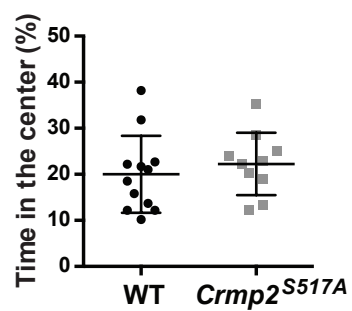**C**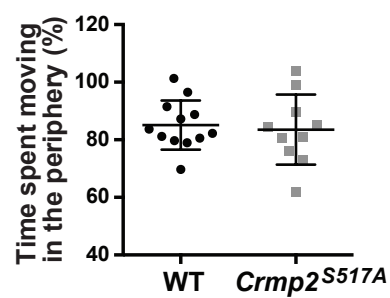**D**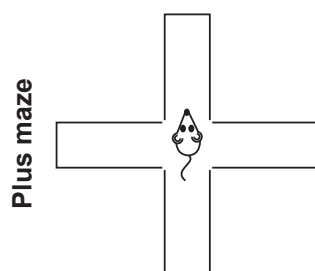**E**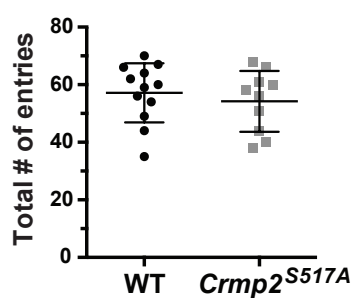**F**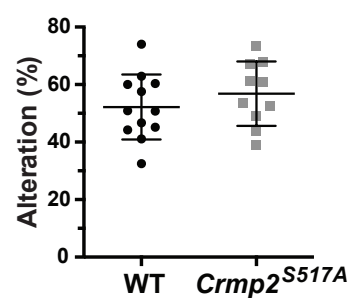

Supplementary Figure S7.

Supplement: Figure S7: Behavioural characterisation of Crmp2S517A mice [file rsob190192supp5.pdf]
