# Supplementary material for: Loss of CRMP2 O-GlcNAcylation leads to reduced novel object recognition performance in mice
Source: Open Biol. 2019 Nov 27;9(11):190192. doi: 10.1098/rsob.190192 (PMC6893399; doi:10.1098/rsob.190192)
Supplement: Table S1 [file rsob190192supp6.pdf]

A

GlcNAcstatin G	Timepoint (mins)	Mouse 4 blood conc. (ng/mL)
	15	150
	30	177
	60	1425
	120	813
	240	1746
	360	1062
	1260	1326
	1440	1371

B

Mouse	Time (mins)	Average blood conc. (ng/ml)	Average brain conc. (ng/ml)	Brain:Blood Ratio
4	1440	1371	99	0.07

C

Vehicle	Timepoint (mins)	Mouse 1 blood conc. (ng/mL)	Mouse 2 blood conc. (ng/mL)	Mouse 3 blood conc. (ng/mL)	Average blood conc. (ng/mL)
	1440	0	0	0	0

GlcNAcstatin G	Timepoint (mins)	Mouse 5 blood conc. (ng/mL)	Mouse 6 blood conc. (ng/mL)	Mouse 7 blood conc. (ng/mL)	Average blood conc. (ng/mL)
	1440	1374	969	1074	1139

Supplementary Table S1.
