# Supplementary material for: Loss of CRMP2 O-GlcNAcylation leads to reduced novel object recognition performance in mice
Source: Open Biol. 2019 Nov 27;9(11):190192. doi: 10.1098/rsob.190192 (PMC6893399; doi:10.1098/rsob.190192)
Supplement: Table S2 [file rsob190192supp7.pdf]

#	Genotype	Age (days)	Sex	Phosphopeptides Detected	
				Phosphosites on Singly Phosphorylated Peptides	Phosphosites on Doubly Phosphorylated Peptides
1.	WT	46	F	-	-
2.	WT	46	F	-	-
3.	S517A	46	F	-	-
4.	S517A	46	F	Ser522	-
5.	WT	45	F	-	-
6.	WT	45	F	-	-
7.	S517A	45	F	Ser522	-
8.	S517A	45	F	-	-
9.	WT	182	M	Thr509, Thr514, Ser518	Ser518 and Ser522
10.	WT	182	M	Thr509, Thr514, Ser518	-
11.	WT	182	M	Thr509, Thr514, Ser518	Ser518 and Ser522
12.	S517A	182	M	Thr509, Thr514, Ser518	-
13.	S517A	178	M	Thr509, Thr514, Ser518	-

Supplementary Table S2.
