# Supplementary material for: Loss of CRMP2 O-GlcNAcylation leads to reduced novel object recognition performance in mice
Source: Open Biol. 2019 Nov 27;9(11):190192. doi: 10.1098/rsob.190192 (PMC6893399; doi:10.1098/rsob.190192)
Supplement: Table S3 [file rsob190192supp8.pdf]

A

Source	Type III Sum of Squares	df	Mean Square	F	Sig.	Partial Eta Squared	Noncent. Parameter	Observed Power
Intercept	137792.935	1	137792.935	15757.836	.000	.999	15757.836	1.000
Genotype	96.413	1	96.413	11.026	.005	.441	11.026	.870
Sex	1192.314	1	1192.314	136.352	.000	.907	136.352	1.000
Genotype * Sex	5.442	1	5.442	.622	.443	.043	.622	.114
Error	122.422	14	8.744					

B

Age (weeks)	Two-tailed P value	P value summary	Difference (%) mean	SD	SEM	N (pairs)
12	0.0082	**	-3.855	3.780	1.140	11
16	0.0003	***	-5.552	3.386	1.021	11
20	0.0001	***	-5.989	4.055	1.084	14
24	0.0007	***	-5.329	4.028	1.117	13

Supplementary Table 3.
